# Improving clinical research and cancer care delivery in community settings: evaluating the NCI community cancer centers program

**DOI:** 10.1186/1748-5908-4-63

**Published:** 2009-09-26

**Authors:** Steven B Clauser, Maureen R Johnson, Donna M O'Brien, Joy M Beveridge, Mary L Fennell, Arnold D Kaluzny

**Affiliations:** 1 Applied Research Program, Division of Cancer Control and Population Sciences, National Cancer Institute, Bethesda, Maryland, USA; 2 Office of the Director, National Cancer Institute, Bethesda, Maryland, USA; 3 Community Healthcare Strategies LLC, New York, New York, USA; 4 Clinical Research Program Directorate, SAIC-Frederick, Inc., Frederick, Maryland, USA; 5 Sociology and Community Health Departments, Brown University, Providence, Rhode Island, USA; 6 UNC Gillings School of Global Public Health, University of North Carolina at Chapel Hill, North Carolina, USA

## Abstract

**Background:**

In this article, we describe the National Cancer Institute (NCI) Community Cancer Centers Program (NCCCP) pilot and the evaluation designed to assess its role, function, and relevance to the NCI's research mission. In doing so, we describe the evolution of and rationale for the NCCCP concept, participating sites' characteristics, its multi-faceted aims to enhance clinical research and quality of care in community settings, and the role of strategic partnerships, both within and outside of the NCCCP network, in achieving program objectives.

**Discussion:**

The evaluation of the NCCCP is conceptualized as a mixed method multi-layered assessment of organizational innovation and performance which includes mapping the evolution of site development as a means of understanding the inter- and intra-organizational change in the pilot, and the application of specific evaluation metrics for assessing the implementation, operations, and performance of the NCCCP pilot. The assessment of the cost of the pilot as an additional means of informing the longer-term feasibility and sustainability of the program is also discussed.

**Summary:**

The NCCCP is a major systems-level set of organizational innovations to enhance clinical research and care delivery in diverse communities across the United States. Assessment of the extent to which the program achieves its aims will depend on a full understanding of how individual, organizational, and environmental factors align (or fail to align) to achieve these improvements, and at what cost.

## Background

Oncology, like many other medical specialties, is in an era of profound change. The emergence and implications of genomics, proteomics, immunology, and synthetic biology, to name a few fields, will affect the way science is practiced and the way health care is provided [1]. Similarly, research and service delivery capacity to support these changes also will be challenged to ensure that beneficial innovations reach all cancer patients who need them. Meeting this dual challenge requires a reconfiguration involving both research and service delivery in many communities throughout our nation. One such initiative designed to address these challenges is the pilot of the National Cancer Institute (NCI) Community Cancer Centers Program (NCCCP).

The objective of this paper is to describe the NCCCP pilot and the evaluation designed to assess its role, function, and relevance to the research mission of the NCI, as well as its contribution to improving patient care in a non-profit community hospital setting. The program itself is viewed as an organizational innovation and its evaluation as an effort to map the factors that facilitate or impede its ability to meet objectives within a community environment. The evaluation presents a unique opportunity for NCI to focus on program evolution to assess proof of concept as well as on specific indicators of program improvement to assess proof of performance.

We begin by describing the developmental trends that provide the context and rationale for the NCCCP pilot. We then describe the conceptual framework used to organize the evaluation for the NCCCP. This framework, together with the NCCCP objectives and components, define the key analytical questions underlying the implementation and sustainability of the program. The paper ends with a discussion of the implications for the research agenda of the NCI within a changing service delivery environment.

## Discussion

### The emergence of the NCCCP

Two developmental trends within the larger environment provided the rationale for the NCCCP initiative -- NCI's growing commitment to reconfiguring clinical research and the need to improve access to state-of-the-art cancer care in community settings.

### Reconfiguring clinical research

In 2002, the National Institutes of Health (NIH) launched the NIH Roadmap [2]. The roadmap commitment to 're-engineering the clinical research enterprise' has significant implications for quality and safety, and promotes the development of public-private partnerships to transform new scientific knowledge into tangible benefits that can ensure improved cancer care. In 2004, the NCI launched the Clinical Trials Working Group [3] as a means of restructuring and improving the administration of the NCI-sponsored clinical trials program within academic-based and community settings. NCI published its strategic plan in 2006, outlining the need to improve research and its application to improved care delivery throughout the cancer continuum [4].

The NCCCP responds to these initiatives through its emphasis on establishing new partnerships of research and care delivery with organized patient communities, community-based health care providers, and academic researchers. Both the NCI strategic plan [4] and the NCCCP emphasize the need to build better integrated networks of academic centers linked to a qualified body of community-based health care providers who serve large groups of patients and who are interested in working with the research community to quickly develop, test, and deliver new interventions.

### Improving access to state-of-the-art cancer care

Clinical research and care delivery have entered a new era involving an increasing amount of economic, service, and research activity across, rather than within, the boundaries of traditionally defined organizations. Evidence suggests that cancer patients diagnosed and treated in a setting of coordinated multi-specialty care and clinical research are more likely to receive state-of-the-art care [5-7], and for an increasing number of conditions, experience improved survival and enhanced quality of life. [8] Optimal care for cancer patients today requires a focus on the full continuum of cancer care, including risk assessment, prevention, screening, treatment, follow-up care, palliative care, and appropriate end-of-life care [9]. Many of these services are often beyond the scope and reach of discrete oncology practices, as well as existing individual community providers [10]. The resulting fragmentation challenges the provision of coordinated multi-disciplinary care and easy access to clinical trials [11] within a community setting.

This is particularly evident for racial/ethnic minorities, people of lower socioeconomic status, residents of rural areas, and members of other underserved populations who face an unequal burden of cancer [12]. Although state-of-the-art care is available through the NCI network of cancer centers and programs, it is estimated that fewer than one in eight patients is admitted to academic medical centers in the US, and most new cancer cases continue to be treated in hospitals and physician offices located close to the patient's home [13]. A fragmented system of care remains a major obstacle to realizing the promise of emerging science and translating clinical research into clinical practice.

### Theoretical basis for the NCCCP evaluation: Implementation stages within nested layers of organizational and environmental factors

The evaluation of the NCCCP pilot incorporates elements of both formative and summative evaluation research and requires an interdisciplinary, recognized, theoretical framework for organizational change, as well as a mixed methods approach using both qualitative and quantitative data collection strategies. The NCCCP evaluation is an unprecedented initiative for the NCI, given its focus on changes in cancer service delivery and research capacity at the community level, its assessment of multiple levels of analysis across multiple timeframes, and its recognition of differing sets of 'initial conditions' across the various sites.

In order to capture the essential elements of organizational change/innovation adoption and implementation over time as well as multiple levels of influence on that developmental process, we have combined two major conceptual models from organizational theory: the innovation life-cycle model, which emphasizes stages of implementation [14,15], and recent versions of institutional theory applied to healthcare organizations [16-18]. Stage models of implementation focus our attention on the process of implementation as it unfolds over time, with sequences of different activities and organization-building. Institutional theory focuses attention on assessing, understanding, and tracking both material-resource factors within each site's environment (markets, technologies, and industry structures), and institutional pressures (cognitive and normative expectations, legal structures, governance systems. [18]. Institutional theory also includes an emphasis on structures of connection or linkage between organizations, as strategies to control access to resources, confront institutional constraints, and reduce environmental uncertainty [19,20].

**Figure 1:**
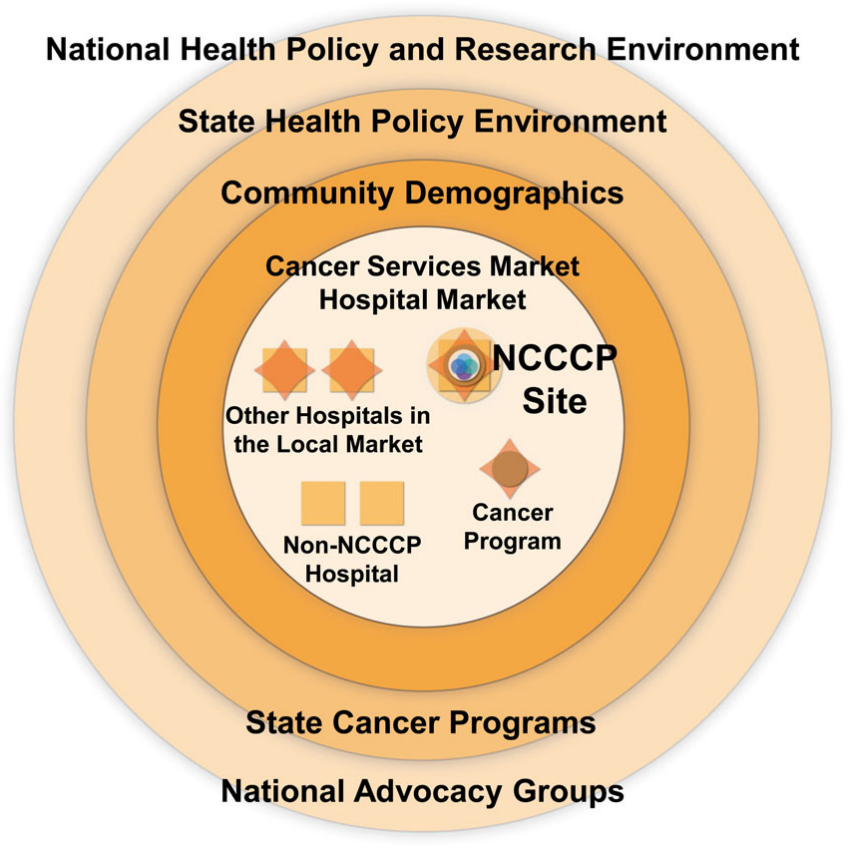
**Environmental Layers**.

**Figure 2:**
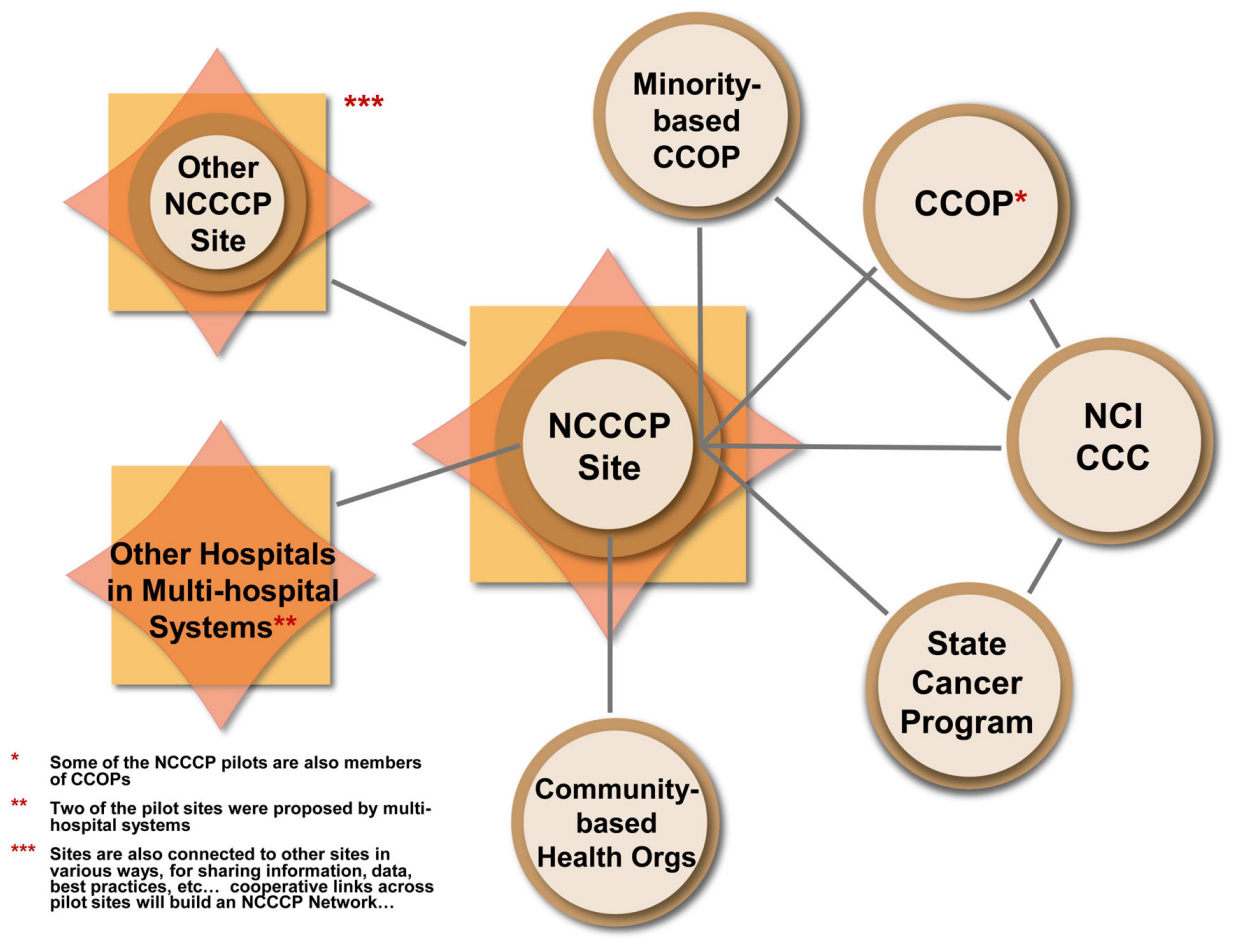
**Important Local and Extra-local Linkages**.

Figure 1 presents a schematic of the basic unit of each demonstration project (NCCCP site located within a cancer program that is part of a community hospital) surrounded by several layers of environmental influences. These layers include the local community and its configuration of patient demographics, the local hospital and cancer services markets, state level policy groups, advocacy organizations and cancer plans/programs, national level policy stakeholders, advocacy groups, medical societies, and federal funding programs. Figure 2 illustrates the types of linkages each pilot could be embedded within at the outset of the NCCCP, or is likely to develop, at both the local level (to other hospitals, community based organizations, and local NCI programs such as comprehensive cancer centers and community clinical oncology programs (CCOP)), and regionally or nationally (state cancer programs, NCI programs).

Using stage models of innovation, the structure, functioning, and performance of the NCCCP pilot can be conceptualized as a process of organizational innovation, unfolding within a multi-layered context of environmental effects that will influence how the pilot sites develop over time. This type of innovation is typically defined as any technology or practice that an organization uses for the first time regardless of whether or not other organizations have previously used the practice or technology. The NCCCP involves a variety of organizational innovations at various phases of implementation. These well-documented phases [14,15] include: initial assessment by relevant personnel within the implementing organization; assessment of readiness for change and the 'fit' between the innovation and organizational values; actual implementation; and, finally, assessment of effectiveness and sustainability. Each NCCCP pilot site is currently engaged in the initial phases of its implementation, assessing and defining the innovation within the cultural context of the implementing organization, developing infrastructure, and building linkages and relationships for program performance.

### Public-private partnerships to integrate research and service delivery in diverse community settings

The objective of the NCCCP pilot is to test a public-private partnership that is designed to bring state-of-the-art cancer care (including early-phase translational science) to all cancer patients in the community, using linkages with other NCI-sponsored research programs (*e.g*., CCOP, Community Networks Program, Cancer Centers Program). It was originally designed to address four key goals: enhance community cancer center infrastructure and resources to address health disparities and improve access to evidence-based cancer care for underserved populations; improve the research infrastructure in community settings by supporting increased participation in clinical trials (especially early-phase trials); encourage the adoption of electronic medical records for care delivery and research, and integrate these research activities with the cancer biomedical informatics grid (ca-BIG^R^); and assess the feasibility of standardized collection of biospecimens for NCI-sponsored research (*e.g*., the cancer genome atlas).

Within each site, activities are thus organized around four core components: reducing disparities in cancer care; increasing the number of patients enrolled in clinical trials; enhancing the site capacity in information technology; and enhancing the capacity for the site to collect, store, and analyze biospecimens. All of these activities support expansion of the research focus of the pilot organizations, and in each site the NCCCP is located within a cancer program embedded within a community hospital.

**Figure 3:**
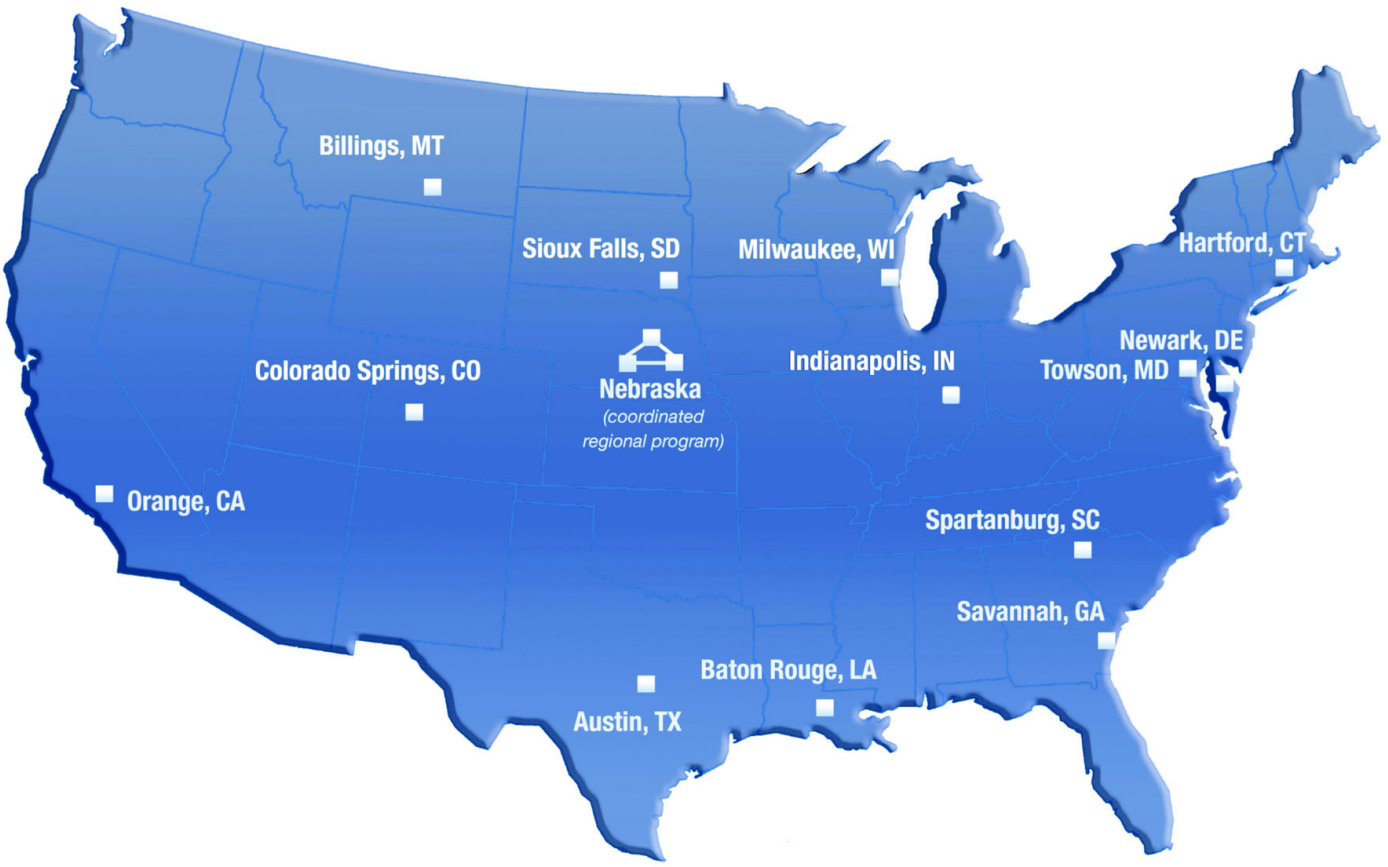
**Map of the NCI Community Cancer Centers Program Sites**.

As illustrated in Figure 3, the pilot is composed of ten geographically distributed non-profit community hospital-based cancer centers that were competitively selected. The ten sites include two multi-hospital systems, one of which has three and the other five affiliated hospital cancer centers. A total of sixteen community cancer centers are included in the pilot. The multi-hospital systems were included to provide a comparison with free-standing community hospitals and to assess whether participation within these systems accelerates diffusion and implementation of various program components among system hospitals [21,22].

Table 1 presents selected site statistics demonstrating the range and scope of the participating hospitals. In 2006, prior to selection as pilot sites, the selected sites served a total population of 12 million people and provided care to 26,000 patients. The sites represent a variety of community settings, with a range of organizational models, expertise, and geographies serving different racial, ethnic, and socio-economic groups. However, all NCCCP awardees met the pilot baseline criteria established in the request for proposals (see Appendix 1). Building from this base, yet recognizing that each site uses different approaches to address the needs of its respective community, all sites will focus on improvement projects as deliverables for the pilot and will be assessed with appropriate metrics derived from the combined conceptual models (see Table 2). The NCCCP evaluation provides an opportunity to assess both the ongoing process changes within a community context at multiple levels of analysis and, within the three year life of the pilot, assess the impact on selected outcome variables.

**Table 1:** Estimated Total Number of Cancer Diagnoses and Patients Treated in 2006 by Study Site

		**Number of Cancer Patients Treated**^2^					
		
**Study Site**	**Total Population** **Service Area**^1^	**Breast**	**Colorectal**	**Prostate**	**Lung**	**Other**	**TOTAL**
Billings Clinic, Montana	283,828	186	133	207	150	753	1,429

Christiana Hospital, Delaware	571,322	533	258	367	447	1258	2,863

Hartford Hospital, Connecticut	1,054,456	544	217	479	280	1075	2,595

Our Lady of the Lake Regional Medical Center, Louisiana	672,319	362	250	365	539	1075	2,591

St. Joseph's/Candler, Georgia	385,242	227	124	87	253	366	1,057

St. Joseph's, Orange, California	2,432,932	385	147	118	149	728	1,527

Sanford USD Medical Center, South Dakota	489,576	187	147	139	177	586	1,236

Spartanburg Regional Hospital, South Carolina	353,757	244	136	193	253	553	1,379

Ascension Health, based in Missouri: St. Vincent Indianapolis Hospital, Indianapolis, Indiana	1,951,252	573	219	182	287	1934	3,195

Columbia St. Mary's Hospital, Milwaukee Wisconsin	685,066	417	157	227	169	692	1,662

Seton Family of Hospitals, Austin, Texas	1,544,670	371	171	58	300	1132	2,032

Catholic Health Initiatives, based in Colorado: Penrose-St. Francis Health Services, Colorado Springs, Colorado	477,263	208	156	197	110	552	1,223

St. Joseph Medical Center, Towson, Maryland	633,814	205	128	155	124	463	1,078

CHI Nebraska coordinated regional program: Good Samaritan Hospital, Kearney, Nebraska	205,994	87	67	95	77	227	553

St. Elizabeth Regional Medical Center, Lincoln, Nebraska	256,939	215	114	39	87	317	772

St. Francis Medical Center, Grand Island, Nebraska	106,724	104	106	82	59	208	559

**TOTAL**	12,105,154	4848	2530	2990	3461	11919	25,751

^1^**Total Population Service Area**: Data from the 2000 US Census that was updated in 2007 by Claritas, Inc. and purchased from Thomson Healthcare by the National Cancer Institute. Copyright © 2007, Claritas Inc., Copyright © 2007 Thomson Healthcare. ALL RIGHTS RESERVED. Provided by the National Cancer Institute's Cancer Information Service (1-800-4- CANCER)

^2^**Number of Cancer Patients Treated**: Total number of new cancer cases seen at the hospital and the cancer center combined in 2006 based on tumor registry data.

**Table 2:** NCCCP site deliverables and evaluation metrics

**Area**	**Deliverable**	**Metrics**
**Clinical** **Trials**	Increase clinical trial accrual including a specific focus on: • accrual of underrepresented and disadvantaged patients • accrual to all clinical trials including treatment, prevention, and behavioral trials with specific focus to increase accrual to multi-modality trials and NCI-sponsored trials • increase the capability to offer phase II trials and develop protocols for appropriate referral of patients for phase I trials to NCI-designated cancer centers or academic medical research institutes	Track accrual overall and for underrepresented patients • NCI trials • Early phase trials • Linkages with other NCI clinical trials programs (*eg*., Community Clinical Oncology Programs (CCOPs)) • Referrals to NCI-designated cancer centers or academic medical research institutes for Phase I trials Track participation in clinical trials and research activities of NCI funded Cooperative Groups such as: CALGB, ECOG, SWOG, RTOG, NSABP, GOG

**Healthcare** **Disparities**	Demonstrate a documented improvement in health screening activities and outreach to community members including a specific focus on underrepresented and disadvantaged populations Implement a policy that all patients who are screened will be treated with appropriate follow- up care Link with NCI disparities programs (*eg*., Community Networks Program, Cancer Information Service) Increase partnering with local, state, and national community organizations, government and non- government Expand patient navigation	Track screening activities by disease site (*eg*. breast, colon) and focus on underrepresented and disadvantaged populations Track efforts to consistently collect race and ethnicity data. Confirm adherence to screening and treatment policy Track linkages Track number, type, and goals of partnerships Track expanded staff and resources for navigation

**Information** **Technology**	Recommend IT infrastructure requirements, necessary interfaces, and applicability of specific components of caBIG^R^for community hospital settings Implement and integrate electronic health records	Complete individual detailed analysis and report Track implementation of EHRs

**Biospecimens**	Recommend the necessary infrastructure requirements, policies and procedures, cost, and other implementations issues, for biospecimen collection and storage, required for implementation enabling community hospitals to participate in biospecimen initiatives	Complete individual detailed analysis and report

**Quality of** **Care**	Increase Multi-disciplinary (MDCs) care disease-site-specific committees and clinics Increase use of evidence-based guidelines, standards and protocols (*eg*., NCCN, ASCO). Participate in a disease specific Quality of Care study Expand genetics and molecular testing Develop cancer center specific medical staff 'conditions of participation' that will be locally determined requirements to insure that those who provide care as cancer center physicians practice in a manner that is consistent with the patient care, quality, research, and community outreach goals of the NCCCP cancer center	Track number and type of MDCs Track number and type of guidelines. Document improved compliance with guidelines Participate in NCCCP pilot Commission on Cancer quality of care study to measure improvements in breast and colon cancer treatment Track components of the genetics program that are offered on site or through referral over time Adopt and implement 'conditions of participation'

**Survivorship**	Expand survivorship and palliative care programs	Provide patient treatment summary to patients. Track new or expanded survivorship and palliative care programs/activities

### Linking the conceptual model to evaluation of the NCCCP model and sites

Building from our combined theories, a number of hypotheses have been developed to guide the evaluation design and help assess NCCCP outcomes of program-specific goal accomplishments, and sustainability/institutionalization over time. As an example of the expected influence of important variables of environmental context on the success of the NCCCP pilot, the following hypotheses were developed connecting variation in levels of hospital competition and cancer services competition on the likelihood of NCCCP sites success in achieving program goals:

Hypothesis one: Pilot sites embedded with community hospitals that are in relatively weak market positions (*i.e*., not the dominant or major player) are less likely to successfully implement and achieve the aims of the NCCCP (such as improve clinical trial accrual rates, offer more multidisciplinary care, or have higher use of evidence-based guidelines) than pilot sites embedded within community hospitals that are dominant within their local markets.

This hypothesis recognizes both the important influence of the community hospital setting on achievement of program goals (and direct support of the site by hospital management), and market influences that might constrain community hospital support of NCCCP activities. The more competitive the local hospital market, the less likely a host-site is to have flexible resources available to support NCCCP activities.

Hypothesis two: Pilot sites embedded within highly competitive local cancer services markets (multiple cancer programs, NCI-designated cancer centers, and/or CCOPs) are less likely to successfully implement and achieve the aims of the NCCCP than pilot sites embedded within less competitive local cancer services markets.

This hypothesis focuses on the specialized market for cancer services within the community, again recognizing that a competitive environment often constrains organizational focus and resources to 'the bottom line,' and away from innovative programming. However, competition for scarce resources can sometimes push organizations to connect cooperatively to other actors through strategic alliances to reduce uncertainty. Further, the development of strategic linkages to other cancer service providers may be more advantageous at different stages of implementation, depending upon other characteristics of context, or histories of pre-existing linkages [23].

**Figure 4:**
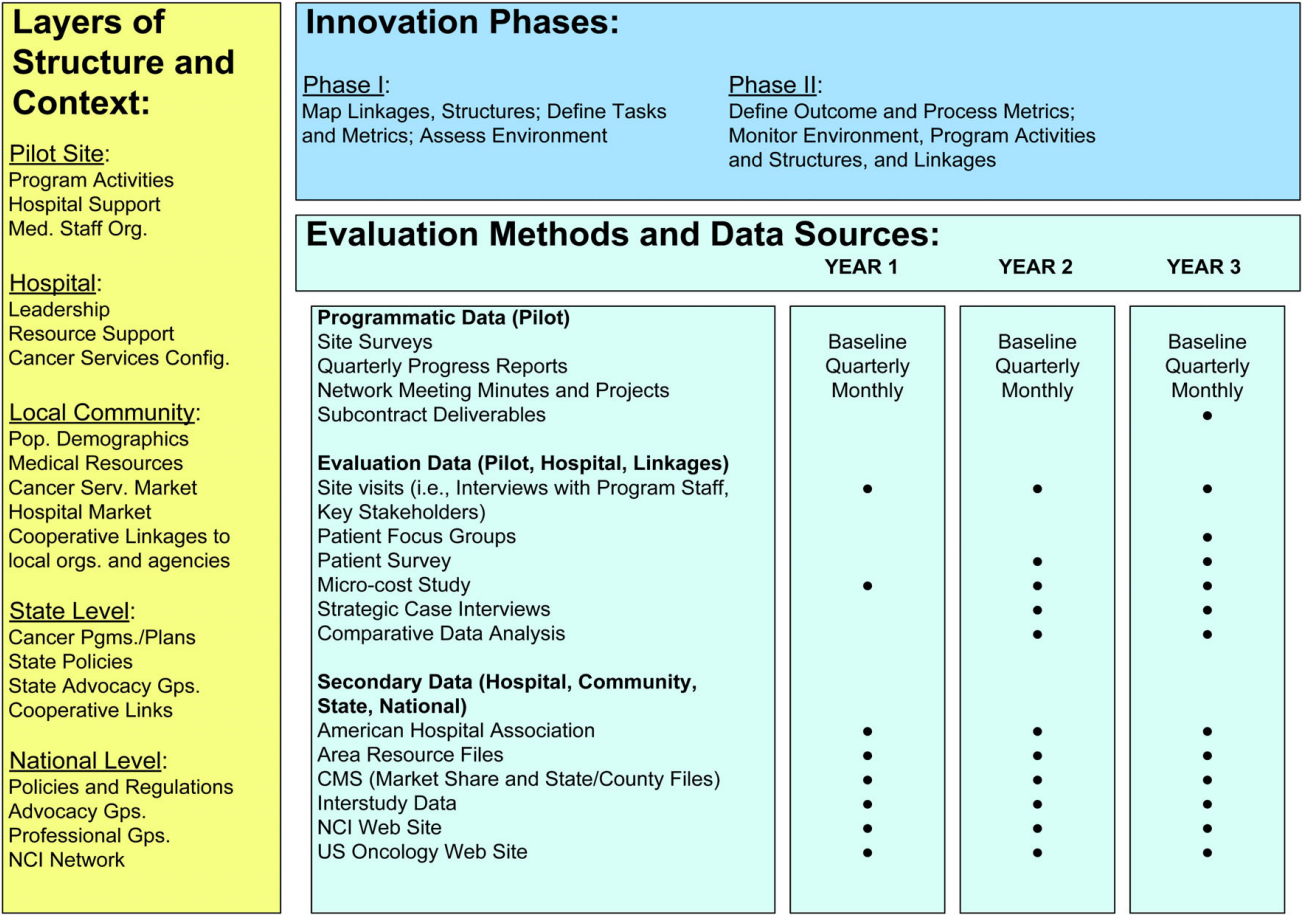
**Innovation Phases and Levels of Observation**.

The application of our combined theoretical perspectives requires an evaluation design that brings into focus the ongoing structures and processes within the participating organizations and the environment within which they function, and how these structures and processes evolve over time. The evaluation involves a phased longitudinal assessment of the pilot program over a three-year period. Figure 4 presents a matrix combining the stages of innovation implementation (along the horizontal) with various layers of site structure and environmental context (arrayed along the vertical). Within the matrix are indicators of when observations will be taken on various variables. The 'metrics' found in Table 2 correspond to outcome- and process-related performance indicators that are linked to evaluation hypotheses, such as the two examples above.

### Phase one

The initial phase of the evaluation will map inter-organizational relationships within programs to project activities as well as the emergence of organizational linkages across pilot sites and between pilot sites and external organizations. Documenting these organizational relationships involves the development of what Miles and Huberman [24,25] have labeled 'context charts' that locate each pilot site in its own web of reporting relationships, formal and informal communication structures, and administrative structures. Context charts are similar to customized organizations maps, which graphically represent the interrelationships among the roles, groups, and organizations that make up the intra- and inter-organizational context of each site (see Figure 2). This kind of map is important not only for describing and understanding each site within its local intra- and inter-organizational context, but also for tracking over time how well the program becomes embedded within its organizational environment.

### Phase two

Building on the initial assessments, evaluation metrics will be identified that correspond to site-specific work plans in the core components of the program. Special attention will be given to the appropriateness of the metrics for the evaluation questions, and the feasibility of site implementation and data collection in a manner consistent with cross-site evaluation.

Based on the information collected in these two phases, a plan has been created that outlines in detail the qualitative and quantitative methods, measures, and data collection protocols that will guide the formal evaluation of the pilot program. This evaluation will involve both a process assessment and an impact assessment of the implementation, operations, and performance of the NCCCP pilot sites. Assessing change in accrual, practice patterns and adherence to evidence-based guidelines within the limited three-year time frame of the pilot is a challenge. However, other community-based initiatives have documented significant changes within a similar time frame including increased accrual with the launch of the minority based - CCOP [26] as well as changes in clinical practice patterns attributed to various hospital-based quality improvement projects [27,28].

The process assessment will evaluate the implementation experience of the specific NCCCP pilot sites, and in subsequent data collection activities through individual site assessments and comparative research. It also will assess the program improvements, best practices, and the sites' relationships to NCI-designated cancer centers and other community and national program resources. These process assessments will be supplemented with information from patient and family member focus groups and a cross-site patient survey to elicit the performance of the program from the patients' and families' experience.

The impact assessment will address a traditional set of evaluation objectives that should be fully answered and understandable once the early stages of the NCCCP and the pilot formative stages are clearly understood. The following evaluation questions will guide that analysis. They are in large part derived from the conceptual model described above:

1. What changes in practice patterns, trial accrual, and adherence to evidence-based practice are attributable to the NCCCP pilot?

2. What factors (*e.g*., NCCCP pilot activities, related hospital organizational factors, local medical staff relationships, NCI partnership, NCCCP network collaborations) are associated with these changes?

3. What are the patient and/or family experiences associated with these changes?

4. What program changes and associated program elements of the NCCCP pilot are likely to be sustained or institutionalized within the existing sites? Which elements appear to be dependent on unique attributes of individual sites?

5. What is the potential for replicating these results in similar community-based cancer programs that did not participate in the NCCCP pilot? What factors (*e.g*., funding, expertise, program infrastructure, program relationships within the hospital authority and resource structure, policy issues, NCCCP network collaborations) are necessary to facilitate the expansion of the NCCCP to other community-based cancer programs?

### Assessing cost of the NCCCP

A special component of the evaluation will be an assessment of the cost of the program. As a public-private partnership, the NCCCP pilot involves significant co-funding to achieve its aims. NCCCP pilot sites have committed at least $47 million to supplement NCI funding over the three-year pilot, matching $3 for every $1 provided by NCI. A critical evaluation question is what the 'true' cost of the NCCCP model is, and how realistic it is for the current pilot sites to sustain these program activities or any future pilot site to replicate the pilot experience. Start-up and regular operating costs associated with the NCCCP pilot will be evaluated. Micro-cost analyses will include labor costs, supplies, equipment, and consulting or contract costs associated with organizational support for the NCCCP pilot. Appropriate efforts will be made to collect and allocate information on staff time spent across specific pilot activities. For the additional sources of external funding, or substantive in-kind contributions that sites contribute to the pilot activities, other external funding and the difference between total external and internal (in-kind) funding will be tracked.

The cost assessment will include a macro-cost component (Dalton K: Business Case Studies, Addressing the Strategic Case for Site Participation, submitted to NCI on February 28, 2008) that distinguishes between what Leatherman *et al*. [29] have termed the business case, the economic case, and the social case for quality improvement initiatives. The social case can be made if the intervention can be shown to improve quality, health status, and access to care or some other socially desirable outcome. The economic case exists if discounted financial benefits of the intervention are greater than discounted costs, even if this occurs only over a long time horizon. The business case, however, requires not only a positive financial return, but also that the potential for benefits accrue to the same entity that makes the program investment, and that benefits occur within a time frame that is short enough to be valued by that entity. While evidence suggests that health care organizations have challenges in achieving and sustaining social, economic, or business returns in the context of program improvement initiatives [29], we hypothesize that it is the alignment of these cases in the context of program policy and implementation, rather than other characteristics of the organizations themselves, that predict these results. This assessment will be valuable in assessing the longer-term feasibility and sustainability of the NCCCP, and what changes in the program model might be necessary to better align NCI goals with the incentives and constraints facing community cancer center programs.

## Summary

NCI increasingly recognizes the critical role that multi-level systems interventions will play in improving health, both in clinical research and in clinical care. Federal research institutions are scrutinized and criticized for the limited existing initiatives that facilitate a rapid translation of research findings into clinical community and public health practice. The NCCCP, initiated as a pilot program, represents the implementation of a major systems-level set of organizational innovations to enhance clinical research and care delivery in diverse communities across the US. Its success will depend, in large part, on inter- and intra-organizational collaboration and cooperation in multiple spheres. Assessment of the extent to which the program achieves its aims will be challenging in a three-year pilot, and will depend upon a full understanding of how individual, organizational, and environmental factors aligned (or failed to align) to achieve these improvements, and at what cost. Current theories of organizational innovation and change provide useful perspectives to guide evaluation design and to help identify why certain results were achieved or not achieved, and options to enable community cancer centers to build on this experience in their efforts to work with NCI to deliver research and evidence-based care to cancer patients where they live.

## Competing interests

The authors declare that they have no competing interests.

## Authors' contributions

All authors contributed to the design, coordination, drafting and review of the manuscript. SBC, ADK, DMO and MLF contributed to the manuscript conceptualization. MJ, JMB, and DMO prepared the tables for the manuscript, as well as figure 3. MLF conceptualized and developed Figures 1, 2 and 4, and led revisions of the manuscript following review. JMB contributed to the graphics of figures 1, 2 and 4. All authors read and approved the final manuscript.

## Appendix 1: NCCCP baseline criteria

• Discrete cancer center with medical, surgical, and radiation oncology under one administrative and medical structure

• A strong oncology practice leadership group committed to providing vision, oversight, and plans for growth and research support

• Physician director with cancer expertise

• A clinical trials program with at least 25 patients enrolled annually

• At least 1,000 annual new cancer cases

• Cancer screening programs

• Multi-disciplinary cancer committees

• Use of evidence-based clinical guidelines

• Patient navigation services

• Infrastructure and programs for community outreach to underserved populations and a policy that all patients screened for cancer will receive treatment for cancer

• An electronic health record or implementation plans underway

• Commission on Cancer accreditation

• College of American Pathology, or Joint Commission Accreditation for Laboratory

• Hospital Chief Executive Officer (CEO) support

• Supplemental funding to support the public/private partnership

• No more than $3 million dollars in NCI funding per year
